# Impact of Fortified Whole Grain Infant Cereal on the Nutrient Density of the Diet in Brazil, the UAE, and the USA: A Dietary Modeling Study

**DOI:** 10.3390/children12030384

**Published:** 2025-03-19

**Authors:** Lynda O’Neill, Maria F. Vasiloglou, Fanny Salesse, Regan Bailey, Carlos Alberto Nogueira-de-Almeida, Ayesha Al Dhaheri, Leila Cheikh Ismail, Nahla Hwalla, Tsz Ning Mak

**Affiliations:** 1 Nutrition Sciences, Nestlé Institute of Health Science, Nestlé Research, 1000 Lausanne, Switzerland; maria.vasiloglou@rd.nestle.com; 2 UCD Institute of Food and Health, School of Agriculture and Food Science, University College Dublin, D04 V1W8 Dublin, Ireland; fanny.salesse@ucdconnect.ie; 3 Institute for Advancing Health through Agriculture, Texas A&M University, College Station, TX 77840, USA; regan.bailey@ag.tamu.edu; 4 Department of Medicine, Federal University of Sao Carlos, Sao Carlos 13565-905, Brazil; dr.nogueira@me.com; 5 Department of Nutrition and Health, College of Medicine and Health Sciences, United Arab Emirates University, Al Ain 15551, United Arab Emirates; ayesha_aldhaheri@uaeu.ac.ae; 6 Department of Clinical Nutrition and Dietetics, College of Health Sciences, University of Sharjah, Sharjah 27272, United Arab Emirates; lcheikhismail@sharjah.ac.ae; 7 Nuffield Department of Women’s & Reproductive Health, University of Oxford, Oxford OX1 2JD, UK; 8 Department of Nutrition and Applied Sciences, American University of Beirut, Beirut P.O. Box 11-10236, Lebanon; nahla@aub.edu.lb; 9 Nestlé Institute of Health Science, 21 Biopolis Road, Singapore 618802, Singapore; tszning.mak1@rd.nestle.com

**Keywords:** whole grain, infant cereal, complementary feeding, fortified, disease prevention, Brazil, UAE, U.S.

## Abstract

Background/Objectives: Complementary feeding (CF) influences future health outcomes. The aim of this study was to evaluate the impact of fortified whole grain infant cereal (WGIC), a complementary food, among 6- to 12-month-old infants on the nutrient density of the diet in three diverse settings: Brazil, the United Arab Emirates (UAE), and the US. Methods: Data from the Feeding Infants and Toddler Study (FITS), a collection of dietary intake studies based on 24-h-dietary recalls, from said countries was utilized. Nutrient intakes were calculated for infant cereal (IC) consumers and non-consumers. Diet modeling was applied to IC consumers to substitute their regular fortified IC with WGIC with improved fortifications. The study estimated the average nutrient density, mean adequacy ratio (MAR), and percentage inadequacy of the diet in both IC consumers and non-consumers. Results: The analyses indicated that infants who consumed IC had higher intakes of calcium, zinc, magnesium, iron, and vitamin D in the three countries. Reduced micronutrient inadequacies were observed among IC consumers, particularly in Brazil and the U.S. Diet modeling with WGIC revealed a significantly higher density of choline, magnesium, zinc, iron, fiber, and protein, as well as reduced inadequacies. The MAR was significantly improved in the three countries. Conclusions: This study underscores the potential of fortified WGIC in increasing the nutrient density of the complementary diet. The intrinsic nutrients in whole grain infant cereals (WGICs) significantly enhance the nutrient density of the complementary diet. Given that whole grains play a role in preventing childhood obesity, their inclusion during CF may be crucial.

## 1. Introduction

Diet quality has been recently reported as suboptimal in infants across low- and middle-income countries [1], as well as in high-income settings [2,3,4,5,6,7,8]. As the evidence linking diet quality in infancy with health outcomes in later life is growing, strategies to fill food and nutrient requirements are deemed necessary for the prevention of malnutrition [4,5] and the optimization of long-term health [9]. The CF period, from 6 to 24 months, consists of a gradual transition from a milk-based diet to a wide range of foods and represents a critical window for the provision of essential nutrients [10,11]. Given the high nutrient needs relative to the narrow range of energy needs during CF, foods should be particularly micronutrient dense, especially among older infants aged 6 to 12 months [12]. Indeed, in terms of micronutrient density (the quantity of a nutrient per 100 kcal), the needs of an infant are much higher than at any other life stage, particularly for iron, zinc [13], calcium, and choline [12]. Therefore, 2 servings per day of nutrient-dense foods, such as animal-source foods, are recommended by the American Academy of Pediatrics [10] and the World Health Organization [8] during the CF period.

Evidence suggests that early exposure to nutritious foods with distinct flavors can maximize the probability that children will accept and choose such foods in subsequent years [14,15,16,17]. Thus, dietary patterns and feeding behaviors established in early life tend to persist into later stages of childhood and beyond, influencing long-term health [18,19]. CF therefore represents a prime opportunity to introduce whole grains (WGs) to the diet [19,20,21] considering that WGs are not ubiquitously consumed by adults [22] but are known to confer multiple health benefits across the lifespan [23]. WGs play a significant role in preventing obesity in children due to their high nutritional value, fiber content, and ability to regulate energy intake. Rich in dietary fiber, whole grains promote satiety and help control appetite, reducing the likelihood of overeating. They also have a lower glycemic index compared to refined grains, leading to more stable blood sugar levels and fewer cravings for unhealthy food. Studies consistently show that children who consume more whole grains tend to have a healthier BMI and a reduced risk of obesity. WGs are recommended during CF for various reasons including their fiber and micronutrients levels [24]. Whole grains also contain polyphenolic phytochemicals, such as phenols, flavonoids, and carotenoids (lutein, zeaxanthin, and β-cryptoxanthin) [25]. Emerging evidence indicates that WGIC may be advantageous, not solely for its nutrients and polyphenols, but for its potential beneficial impact on the infant microbiota maturation and diversity [26].

Infant cereals (ICs) are frequently recommended as first foods as they have been shown to enhance iron and zinc intake via fortification [27]. In the U.S., IC consumption is gradually declining while in parallel iron inadequacy is increasing among the infant population [28]. Moreover, while CF patterns and practices vary based on geographical, cultural, and socio-economic diversity, as well as diverging traditions, values, and beliefs [29], many infants globally already consume fortified infant cereals [20]. Dietary intake studies have reported that infants who consume fortified ICs have higher intakes of iron [30] as well as calcium, magnesium, zinc, and vitamin E compared with those that do not [31]. Nevertheless, potassium intakes in infants aged 6–12 months fall short of 90% of the Adequate Intake (AI) at various energy levels, implying the necessity to consume foods such as whole grains, which are generally intrinsically richer in potassium, iron, and many other micronutrients, compared with refined grains [11]. However, most IC products consumed globally are produced with refined grains [20] and there is limited evidence on the comparative effects of WGIC versus traditional IC on nutrient density and overall diet quality in infants.

Despite the importance of introducing nutrient dense foods during this period, many infants globally fail to satisfy their nutrient requirements. According to the Feeding Infants and Toddlers Study (FITS) 2016 data, Bailey and colleagues reported that infants in the US are at risk of dietary inadequacy for vitamins D and E [2]. This study also observed that about 18% of infants experienced inadequate iron intake. Additionally, in a FITS study in the United Arab Emirates (UAE) [32], the authors reported a substantial proportion of the infant population had inadequate intakes of iron and zinc (about 45% and 30%, respectively), while a low proportion had intakes of vitamins A and D above the AI. Although the UAE is an affluent country, it is currently undergoing a nutrition transition [33]. Brazil, which is considered an upper-middle-income country [34], is also undergoing a nutrition transition. Inadequacies in iron, zinc, and vitamins A and D were reported in a systematic review on dietary intake studies in Brazil [35]. Moreover, the extent of said inadequacies was generally greater than in the UAE and the review also found insufficient intakes of calcium and vitamin C. While previous studies have identified nutrient inadequacies, they do not explore innovative solutions like WGIC that may potentially address said inadequacies.

The development of new IC recipes, such as WGIC, is in line with advancements in food science, offering superior, age-appropriate nutrition for infants and addresses the increasing consumer demand for convenient and safe products [36]. Given the important role of fortified IC, the importance of WG in the diet, and the disparities seen in CF around the world, the objective of this study was to evaluate the impact of fortified WGICs on the nutrient density, diet quality, and adequacy among 6- to 12-month-old infants in three countries that differ in terms of economic status, fortification practices, and dietary and cultural practices: Brazil, the UAE, and the U.S. First, we compared nutrient density, diet quality, and percentage inadequacy of IC consumers versus those of non-consumers across countries. Additionally, we simulated the substitution of any consumed fortified ICs with a new WGIC recipe with additional fortifications to evaluate its theoretical impact on the diet of current IC consumers. This research aimed to deliver crucial insights into the impact of fortified whole grain infant cereals on improving nutrient density and dietary quality among infants, thereby filling significant gaps in the existing scientific literature.

## 2. Materials and Methods

### 2.1. Study Population

The Feeding Infants and Toddler Study (FITS) is a collection of cross-sectional nationwide surveys using a 24 h recall method to gather information on dietary intakes and feeding practices [37]. In this current paper we used the FITS data of 6- to 12-month-old infants from Brazil, the U.S., and the UAE. While CF extends from 6 to 24 months, only infants of ages 6 to 12 months were considered as IC consumption sharply declines after the age of 12 months [38]. Thus, the younger age group were the most relevant to the study. Three countries were selected to represent a highly developed Western country (the U.S.), another high-income country with a different culture from the U.S. (the UAE), and a middle-income country with a distinct culinary heritage that includes infant cereals and whole foods (Brazil) [35].

The 24 h recall was used to capture the dietary intake of the infants, and additional questionnaires were administered in each of the countries to supplement the information gathered. A screener questionnaire to identify eligible participants, a recruitment questionnaire with sociodemographic and lifestyle questions, and a feeding practices questionnaire were used in the U.S. and the UAE (e.g., breastfeeding practices, introduction of complementary foods). In Brazil, a household screener, age-specific dietary questionnaires, age-specific general questionnaires, anthropometry measurements, and a record of calls and visits were used. In the three countries, parents or caregivers provided informed consent. Overall, parents or caregivers served as the main study respondents for all modules. All study procedures were approved by the Institutional Review Board of the American University of Beirut, the UAE Ministry of Education, the Human Research Ethics Committee of the United Arab Emirates University, the Dubai Scientific Research Ethics Committee of the Dubai Health Authority, the Research Ethics Committee and Subcommittee of the UAE Ministry of Health and Prevention, and the Research Ethics Committee at the University of Sharjah.

The U.S. data were collected from different states and a detailed report of the FITS 2016 design and methodology was reported previously [37]. Dietary recall interviews were conducted by telephone and administered by trained and certified interviewers using the multiple-pass 24 h recall methodology using the Nutrition Data System for Research (NDSR, version 2015: University of Minnesota, Minneapolis, Minnesota) to convert foods into nutrients. The interviews were conducted with the parent or primary caregiver responsible for feeding the child (i.e., proxy), and a form was provided to assist the proxy in collecting dietary recall data from daycare centers and other caregivers where the child may have spent time away from the proxy reporter.

In the UAE, data were collected between June 2019 and January 2020 from primary health care centers and outpatient clinics at hospitals in three regions where about 85% of the country’s population lives. The sampling method and study design have been documented elsewhere [32]. In the clinic waiting rooms, trained nutritionists contacted parents and caregivers and offered them to participate in the study by outlining its goals and protocol. The nutritionist interviewed the proxy reporter one-on-one in a separate room in the clinics after obtaining their written consent. The participating caregivers filled out a multi-component questionnaire during the interview about sociodemographic and economic characteristics, early-life feeding habits, and food consumption, as well as the use of vitamin and mineral supplements. The multiple-pass 24 h recall approach was used to evaluate dietary intake and the Nutritionist Pro software, (version 5.1.0, 2014, Nutritionist Pro, Axxya Systems, San Bruno, CA, USA) in combination with USDA food composition edited by the American University of Beirut (AUB) for local main dishes, was used for converting foods into nutrients.

Data from Brazil were collected between October 2019 and February 2020 from a convenience sample living in the three most populated regions of Brazil: Northeast, Southeast, and South. The dietary recall module was collected on paper by field interviewers and then entered into the NDSR (version 2019: University of Minnesota, Minneapolis, Minnesota) by trained nutritionists. Dietary recall collection procedures followed the selection of households using random walk procedures, and field interviewers screened households for eligibility. The interviewer then collected the 24 h dietary recall using a tailored dietary recall form and scripts aided by the Food Amount Estimation tools [39].

### 2.2. Diet Modeling

The impact of substituting the current IC intake with that of a WGIC recipe was assessed using three strategies. First, the total daily amount of IC consumed (in grams) in each IC consumer infant was determined, as well as the amount provided by IC for each nutrient based on the nutritional composition of the IC products. Second, the total intakes from ICs were removed from the diet of infants and replaced by the same quantity (in grams) of the new WGIC recipe. Finally, the percentage of those at risk of nutrient inadequacy, using the Dietary Reference Intakes [40] before and after applying the substitution, was calculated for the scenarios. In all cases, IC and WGIC refer to fortified products.

#### Selection of the Recipes

The WGIC recipes used in the modeling scenarios were different for each country to be compatible with current consumption practices and ensure compliance with local regulations [41]. The recipes were developed by selecting the most consumed product per country in the relevant FITS dietary intake survey, including WGs, and adapting the fortification levels to address nutrient gaps observed among IC consumers. This involved adding vitamin D and/or choline according to permitted levels in the three countries. Alongside the inclusion of WG, the recipes had to be adapted to ensure processability and minimize the formation of contaminants to guarantee optimal food safety and quality of the final product are in line with the highest baby food standards. The nutritional composition of the recipes is shown in the Supplementary Materials. Regardless of the recipe, the substitution only involved dry cereal powder, and no reconstitution liquids were substituted or altered in any way.

### 2.3. Reported Nutrients

For the purpose of this study, only data on the major macronutrients and critical micronutrients were reported. Critical micronutrients were defined as those that are frequently lacking in the infant diet: iron, zinc, calcium, vitamins A, D, and folate [42], as well as magnesium, potassium, and phosphorus [43]. Thiamin, niacin, and choline were included as studies have found them to be difficult to achieve when planning complementary diets [11].

### 2.4. Statistical Analyses

Mean nutrient intakes were calculated for IC non-consumers and consumers before and after applying modeling scenarios and were expressed as nutrient density (per 100 kcal of intake). Non-normal data were transformed prior to analysis; the non-parametric Mann–Whitney U test was used to test the difference in nutrient density between IC consumers and non-consumers. Outliers were removed based on an energy intake three standard deviations from the mean intake (1 outlier was removed in Brazil, 14 in the U.S., and 1 in the UAE, representing between 0.9 and 1.5% of the samples).

The National Academies of Sciences, Engineering, and Medicine (NASEM), formerly known as the Institute of Medicine (IOM), used dietary reference intakes (DRI) to compare observed intakes to the reference values for this age group [44,45]. Updated sodium and potassium [43], as well as calcium and vitamin D [46] reference values, were also from NASEM. The U.S. DRIs were regularly applied in studies in Brazil [39] and in the UAE [33] as these countries do not have their own reference values. The estimated average requirement (EAR) cut-point method was used to establish the percentage of infants at risk of inadequate intakes This method was developed by Beaton (1985) [47] and further explored and validated by the IOM [48]. To determine the risk of inadequate intakes both before and after implementing the modeled scenarios, the proportion of infants with nutrient intakes below the EAR was computed.

The Mean Adequacy Ratio (MAR), a diet quality index, was calculated for each individual based on the Nutrient Adequacy Ratio (NAR) per nutrient. The Nutrient Adequacy Ratio (NAR) was capped at 1 (corresponding to 100% adequacy) to ensure that a nutrient with a high NAR could not offset a nutrient with a low NAR. The formulas to compute the NAR and MAR are shown below. The Recommended Daily Allowance (RDA) values were based on the same Dietary Reference Intakes derived from the IOM/NASEM. In the absence of an RDA for a given nutrient, the Adequate Intake was used.

$$\text{NAR}=\frac{\text{Daily intake of nutrient}}{\text{RDA}}\;\;\;\;\text{MAR}=\frac{\sum \mathrm{NAR}\;(\mathrm{each}\;\mathrm{truncated}\;\mathrm{at}\;1)}{\text{number of nutrients}}$$

The Mann–Whitney U test was used to test the differences in mean intakes between groups for the observed intakes. The Wilcoxon rank-sum test was applied to compare before and after the diet modeling. The differences between proportions of inadequacies were assessed using Fisher’s exact test. All *p*-values were adjusted to account for false discovery rates given multiple comparisons [49]. All data were analyzed using R version 4.2.2 and accounted for the sampling framework.

## 3. Results

### 3.1. Descriptive Statistics

Table 1 describes the infant population for each country. The total number of infants aged 6 to 12 months was 108 for Brazil (33 IC consumers, 75 non-consumers), 911 for the U.S. (448 IC consumers, 463 non-consumers), and 72 for the UAE (29 IC consumers, 43 non-consumers). The sample comprised more boys in Brazil (55.6%) and the U.S. (54.1%) and more girls in the UAE (52.8%), but these proportions did not significantly differ across countries (*p* = 0.491). However, the parent’s education level was found to be significantly different (*p* < 0.001), with a majority of parents reaching a high school level in BZ, compared to the U.S. and the UAE where most parents achieved a higher educational level. The U.S. also showed the highest proportion of infants attending daycare (31.4%). The majority of infants were ever breastfed (92.6% in Brazil, 85.4% in the U.S., 93.1% in the UAE) and, on average, CF provided between 40% and 46% of the energy (in the UAE and in Brazil, respectively).

### 3.2. Observed Diet

The comparison of intakes between IC consumers and non-consumers is presented in Table 2 as the nutrient density of the total diet, and in Table 3 as percent inadequacy of selected nutrients. Calcium, zinc, iron, and vitamin D intakes were significantly higher in all countries among infants who consumed IC when compared with those that did not. In Brazil and the U.S., the nutrient density of the diets was higher among IC consumers, except for total fat, choline, and sodium, as well as protein in the U.S. Moreover, the MAR was significantly improved (i.e., closer adherence to recommended intakes) in IC consumers when compared to non-consumers in Brazil and the U.S. The prevalence of inadequacies was also significantly reduced for most studied nutrients in Brazil, and for most nutrients in the U.S except for choline and sodium. Regarding the UAE, the consumption of any IC was associated with a reduction in the density of fat in the diet, but increased that of calcium, iron, zinc, and vitamin D. A decrease in zinc and thiamin inadequacy levels was also observed.

### 3.3. Modeled Diet

Results of the modeling scenario are presented in Table 4 and Table 5, as nutrient density of the diet and percentage of infants at risk of inadequacy, respectively. The modeled intakes of WGIC show a higher density of most nutrients in the diet of infants across the three countries. The most notable changes were the significantly higher levels of choline, magnesium zinc, iron, fiber, and protein after modeling. Additionally, a reduction in risk of inadequacy for choline in Brazil and the U.S. was observed. In the UAE, vitamin A significantly increased, resulting in an overall better diet quality as shown by the MAR. Indeed, the MAR improved in the 3 countries after diet modeling. A reduction in the proportion of infants at risk of inadequacy in magnesium was also observed in the UAE. In the U.S., however, a decrease was also observed in folate and potassium density.

## 4. Discussion

In this study, we assessed the impact of the consumption of fortified IC on fiber and micronutrient density among infants living in three different geographic areas. In all countries, the consumption of any fortified ICs significantly improved the nutrient density of calcium, iron, zinc, and vitamin D intakes. Furthermore, the MAR of IC consumers was greater in the 3 countries, indicating that IC consumption is associated with an overall higher diet quality based on the increase in micronutrient intakes. The impact of fortified ICs was also notable in the UAE as consumers had a reduced risk of inadequacy for iron, zinc, vitamin D, and other micronutrients. However, the MAR of IC consumers was not statistically different compared with non-consumers in the UAE. The non-significance may have been because of the effect size taking into consideration the small number of infants constituting the sample for this study. Nevertheless, the results indicate the benefits of IC consumption and highlight the need for ICs in the infant diet to optimize micronutrient intakes and diet quality. This result agrees with other studies that compared the intakes of IC consumers versus non-consumers [30,51]. Overall, this finding could be interesting for low- and middle-income countries (LMIC) and those countries undergoing a nutrition transition. Indeed, a characteristic of countries undergoing nutrition transition is the simultaneous prevalence of both undernutrition and overnutrition i.e., non-communicable diseases such as childhood obesity [52].

Different cultural contexts may influence CF practices [29,53]. Furthermore, socio-economic status, especially household wealth and maternal education, influence CF practices, particularly in LMIC [53,54]. Maciel et al. found this same association between these two characteristics and the WHO core indicators for infant and young child feeding among low-income communities in Brazil [50]. Furthermore, a systematic review found a positive association between maternal education, household wealth, maternal age, and healthy dietary patterns during complementary feeding [55].

Regarding official guideline in Brazil, the Ministry of Health recommends that CF includes cereals only for lunch and dinner, with an emphasis on one serving in each of these meals, and the options included in the guideline are rice, oats, rye, corn, wheat, and their flours, always unprocessed [56]. A Brazilian national study showed that, between 6 and 12 months of age, 73.9% consumed cereals, but predominantly in the form of homemade “porridge” [57], while another study showed the fundamental contribution of rice and cornmeal as ingredients in the preparation of said porridge. Considering that these porridges are homemade, they usually do not contain additional micronutrients at the levels required by infants. Indeed, a review on infant feeding in Brazil, reported that the major inadequacies were iron, vitamin A, and zinc [58], suggesting a suboptimal diet and a lack of fortified foods and/or dietary supplements. In the U.S., the DGAC recommends that CF includes the consumption of meat or the use of iron and zinc-fortified IC, especially among infants [11]. However, meat is not widely consumed in the U.S. by those under 12 months old and the use of fortified ICs has gradually decreased over time; there was also the low use of iron-containing dietary supplements [28]. Subsequently, an increased risk of iron inadequacy has been observed [28]. Due to the absence of local CF guidelines in the UAE, the Ministry of Health and Prevention have endorsed the WHO recommendations on CF, incorporating slight adjustments to ensure cultural adaptation. Aligned with these recommendations, fortified ICs are consumed in this country and provide a significant contribution to micronutrient intakes [59]. Nevertheless, 55% and 31% of infants fail to meet the requirements for iron and zinc, respectively [32]. Globally, given the narrow range of energy needed during the CF period, it can be challenging to meet nutrient recommendations without the use of fortified products or dietary supplements [27].

Fortification is an important strategy to optimize intakes of micronutrients of public health concern [60]. The WHO mentions that fortification of industrially processed grains is a simple, effective, and inexpensive strategy for reducing micronutrient inadequacies in the general population [60]. In the three studied countries, local fortification policies either mandate or encourage the fortification of grains. In Brazil, a mandatory fortification program ensures corn and wheat flours are fortified with iron and folic acid. The fortification policy in the U.S. is similar as it mandates fortification of corn, wheat, and rice with the same micronutrients, while in the UAE, the government encourages voluntary fortification of wheat flour with calcium, iron, folic acid, niacin, thiamine, riboflavin, and vitamin D [61]. However, in general, most fortification practices are intended for the general population, and not specifically designed to target the high micronutrient requirements of infants. Therefore, the consumption of regular fortified grains is not sufficient for many infants, especially breastfed infants, to achieve their high micronutrient needs. Apart from red meat, only fortified IC can fulfil these requirements of infants, which others have also demonstrated [51]. In addition to the commonly known micronutrients of concern, we noted a current high percent of inadequacy of choline in the 3 studied countries, ranging from 42% among IC consumers in Brazil to 93% among IC consumers in the UAE. Considering the emerging evidence on the role of dietary choline in early-life and long-term neurodevelopmental outcomes [62,63], the intrinsic choline in the whole grains was noteworthy. This intrinsic choline reduced inadequacy to 6% among infants in Brazil, 33% in the U.S., and 76% in the UAE. Choline adequacy can be challenging to achieve during CF, possibly because the richest dietary sources of this vitamin tend to be animal-source foods, such as meat, dairy products, and eggs. Aside from infant milks, animal-source foods, while recommended [8], are not consumed by all infants at this age [64] and are unaffordable in insecure food settings [65]. While developing dietary guidelines for 6- to 12-month-old infants, the USDA applied food pattern modeling to design the optimal diet for CF, which fulfilled all nutrient needs but could not achieve choline (as well as some other micronutrients such as iron) adequacy at the energy levels required by infants [11]. Therefore, choline fortification of infant foods, such as ICs, is justified. Vitamin D was added to the recipes in the UAE and the U.S., which improved the inadequacy risk percentage in both countries. Vitamin D is already added to fortified ICs in Brazil at the maximum level authorized. Indeed, fortification is generally limited to prevent excessive intakes approaching the tolerable upper intake levels (ULs) [51]. However, considering the rise in vitamin D deficiency in the U.S., the Food and Drug Administration (FDA) recently approved the addition of vitamin D to grain-based breakfast foods [66]

Our modeling comparison demonstrated proof of principle that fortified IC is a nutrient-dense food, but also that fortified WGIC is even more nutrient-dense, due to the intrinsic fiber, vitamins, and minerals therein [41]. There is no specific dietary fiber recommendation for infants under 12 months of age from the IOM—now known as the NASEM—nor from the European Food Safety Authority (EFSA) [67]. However, the AAP recommends up to 5g of fiber per day for infants up to 12 months of age [10]. Beyond 12 months, the fiber AI recommended is 14g/1000 kcals, which is extrapolated from adult AI and based on the amount of fiber associated with a reduced risk of coronary heart disease [68]. Considering the median energy requirements of children aged 1 to 3 years, the fiber AI is translated to 19 g/day, which has been challenged as being difficult to achieve [69]. Moreover, dietary intake studies in the countries selected for this study have identified fiber as a shortfall nutrient in young children [2,32,39]. Therefore, the transition to the requirement of 19 g/day at age 1 could be facilitated by the consumption of more fiber-dense foods early in the CF period, such as whole grain, containing IC. Indeed, the 2020–2025 Dietary Guidelines for Americans [11], indicate that whole grains can be introduced in infancy and recommend up to 1 oz equivalent (27 g) per day from the age of 9 months onwards. The consumption of whole grains in early life is believed to help with the maturation and diversity of the infant gut microbiome [70,71] that is critical for many aspects of overall health including immune system development and prevention of colonization of pathogens [72]. Emerging evidence suggests that via the gut–brain axis, the infant microbiota may influence several aspects of brain health and development [73], as well as food intake and risk of obesity [74]

An intervention in Malaysia (GReat-Child™ Trial) focused on incorporating whole grains into the diets of overweight and obese children. The study found that children in the intervention group showed significant reductions in BMI-for-age z-scores, body fat percentage, and waist circumference compared to the control group, suggesting that whole grain consumption can aid in managing childhood obesity [75]. The Whole Grain Intake Study (2001–2012) in the U.S., in which researchers analyzed data from nearly 45,000 children and adults, found that higher whole grain consumption was associated with lower BMI and waist circumference. Despite less than 1% of children meeting whole grain recommendations, those with higher intake had a reduced likelihood of being overweight or obese [76].

The present study identified greater adequacy in magnesium intakes among consumers of WGICs. Indeed, whole grains are an important source of magnesium, which has recently been identified as a micronutrient of concern among the general population globally [77] and increasingly among infants in LMIC [78,79]. Magnesium is critical for regulating nerve and muscle function and consequently has a pivotal role in cardiovascular function. Over-consumption of refined grains and under-consumption of nutrient-dense foods are believed to be responsible for the global increase in rates of magnesium deficiency among all age groups [79]. There are only a few food items known to be intrinsically rich in magnesium, namely nuts, dark chocolate, and whole grains, and of these, only whole grains are appropriate for consumption in significant quantities in the infant diet. Overall, considering the intrinsic concentration of whole grains in magnesium as well as fiber, and the health impacts of these nutrients, whole grains should be emphasized as an important component in the CF period.

Careful management is necessary during whole grain processing to maintain nutrients and bioactive compounds. Using mild heat treatment and maintaining lower moisture content can optimize WGIC processing and help retain these components in whole grains [80]. This modeling study is based on actual recipes, which were designed in accordance with strict regulatory and safety requirements. The challenges of processing WGIC to guarantee safety and quality have been described elsewhere [20]. These recipes were carefully crafted to optimize iron absorption based on a recent clinical trial [81]. The form of iron used in these recipes is ferrous fumarate, which is a particularly well-absorbed from whole grain matrices in the presence of vitamin C at a molar ratio of 2:1 between vitamin C and iron [81].

The present study focused on key micronutrients of concern and highlights the relevance of IC to help bridge nutrient gaps, including fiber, choline, and magnesium, during CF. While data have already been published from the U.S. and UAE studies, this is the first report that analyzed data from FITS Brazil, where we gained new insights into nutrient adequacy in Brazilian infants. Moreover, this is the first study that examined dietary intakes of infants aged 6–12 months and the role of IC in diets in three diverse countries concomitantly. Previous studies on IC have been U.S.-focused [51,82]. Our analyses highlight major differences in diet quality per country as the studied countries are diverse in terms of culture, infant feeding practices, and food fortification strategies. In addition, diet modeling, which was used as the main methodology in this study, is an effective and well-recognized method for evaluating the effects of hypothetical changes to food or beverage consumption in relation to nutrient intakes [83,84,85].

However, some limitations to this study need to be considered. The small sample size in the UAE (n = 79), which occurred as the COVID-19 epidemic struck during data collection. Among the infants in the UAE, there were 29 IC consumers. While the sample size of infants in Brazil was sufficient, subdividing them based on IC consumption status resulted in only 33 IC consumers. Despite this small sample size, some significant differences were observed. In addition, for the purpose of identifying IC consumers, only 1 day of dietary intake was considered as we did not have 2 days for the entire population of 6- to 12-month-olds. As a result, the study only provided a glimpse of the children’s food for one day rather than their typical dietary consumption; but at the group level, the mean of one day is representative of the group. In addition, because supplement use was not included in the current investigation, the total consumption of micronutrients may have been underestimated. Therefore, an isometrical substitution may not be the most realistic approach. Nevertheless, the energy density of refined grains is similar compared to WG, with WG containing slightly more kcal per 100 g [86]. This difference is minimal per serving of IC. Finally, nutritional modeling interpretation should be performed with care as this exercise cannot fully predict how populations would adapt their behavior.

Further research is needed to test additional scenarios such as potential other WGIC recipes or different types of substitutions. An example could be to substitute typically consumed grains with fortified WGICs to explore the impact of partly replacing a staple food with fortified cereals. Studies using longitudinal cohort data to explore the association between WGICs and health outcomes in the long term and in real-life settings are needed to substantiate our findings.

## 5. Conclusions

In conclusion, this study underscores the essential role of fortified infant cereals in enhancing the nutrient density of infants’ diets, thereby emphasizing their significance as complementary foods in addressing micronutrient deficiencies. Furthermore, our modeling analyses indicate that whole grain infant cereals (WGICs) fortified with choline, alongside standard vitamins and minerals, can positively influence fiber, magnesium, and choline intake at the population level. Considering the influence of whole grains on weight gain and obesity in children, incorporating whole grains into CF practices may encourage sustained healthy eating habits.

## Figures and Tables

**Table 1:** Sample characteristics by country.

	Brazil (n = 108)		US (n = 911)		UAE (n = 72)		*p*-Value
	n	%	n	%	n	%	
Gender							
Boys	60	55.6	493	54.1	34	47.2	0.491
Girls	48	44.4	418	45.9	38	52.8	
Age							0.407
6 to 8.9 months	51	47.2	481	52.8	41	56.9	
9 to 11.9 months	57	52.8	430	47.2	31	43.0	
Parent’s education							<0.001
Less than high school	38	35.2	33	3.6	4	5.6	
High school level	59	54.6	163	17.9	29	40.3	
Higher educational level	7	6.5	712	78.2	39	54.2	
Other (special education)	0	0	2	0.2	0	0	
Unknown	4	3.7	0	0	0	0	
Attends daycare							<0.001
Yes	14	13.0	286	31.4	5	6.9	
No	94	87.0	625	68.6	67	93.1	
Ever breastfed							0.032
Yes	100	92.6	778	85.4	67	93.1	
No	8	7.4	132	14.5	5	6.9	
Mean ratio of energy provided by milk/by CF	54/46		59/41		60/40		

**Table 2:** Observed nutrient density of infant diets, according to IC consumption, in three countries.

	Brazil (n = 108)			US (n = 911)			UAE (n = 72)		
Nutrients ^1^	Non-IC Consumers (n = 75)	IC Consumers (n = 33)	*p*-Value ^2,3^	Non-IC Consumers (n = 463)	IC Consumers (n = 448)	*p*-Value	Non-IC Consumers (n = 43)	IC Consumers (n = 29)	*p*-Value
Protein (g)	2.7	3.3	**0.003**	2.6	2.4	**0.006**	2.5	2.6	0.402
Total fat (g)	4.3	3.4	**<0.001**	4.7	4.3	**<0.001**	4.7	4.0	**0.018**
Carbohydrates (g)	13.1	14.2	**0.010**	12.3	13.4	**<0.001**	12.0	13.2	0.088
Dietary fiber (g)	0.7	0.8	0.650	0.7	0.8	**<0.001**	0.5	0.5	0.854
Calcium (mg)	65.0	120.4	**<0.001**	64.9	78.7	**<0.001**	58.6	79.6	**0.006**
Choline (mg)	18.8	16.5	**0.005**	21.0	19.2	**<0.001**	15.3	12.7	0.176
Folate (μg DFE)	16.1	22.5	**<0.001**	15.4	18.1	**<0.001**	19.7	21.2	0.375
Iron (mg)	0.6	2.5	**<0.001**	0.9	2.0	**<0.001**	0.7	1.1	**0.018**
Magnesium (mg)	11.5	14.3	**0.003**	10.7	12.5	**<0.001**	10.6	9.1	0.211
Phosphorus (mg)	48.7	73.2	**<0.001**	47.9	48.9	0.137	46.3	53.7	0.077
Potassium (mg)	142.7	161.9	**0.043**	126.0	136.0	**<0.001**	128.3	127.7	0.532
Sodium (mg)	67.7	78.6	0.051	61.5	44.2	**<0.001**	49.8	45.3	0.375
Zinc (mg)	0.5	0.7	**<0.001**	0.6	0.7	**<0.001**	0.4	0.5	**0.006**
Thiamin (mg)	0.06	0.12	**<0.001**	0.07	0.09	**<0.001**	0.08	0.09	0.261
Niacin (mg)	0.6	1.2	**<0.001**	0.9	1.1	**<0.001**	0.7	0.9	0.077
Vitamin A (μg RAE)	105.8	149.9	**<0.001**	90.0	100.1	**0.002**	89.8	64.2	0.051
Vitamin D (μg)	0.4	1.4	**<0.001**	0.6	0.8	**<0.001**	0.2	0.8	**0.026**
**MAR**	0.809	0.977	**<0.001**	0.840	0.915	**<0.001**	0.808	0.846	0.329

^1^ Numbers expressed per 100 kcal of intake per day [43,44,45,46]; ^2^ *p*-values for Fisher’s exact test, adjusted for first discovery rate (FDR).; ^3^ *p*-values < 0.05 are considered significant and are highlighted in bold.

**Table 3:** Observed percentage of infants at risk of micronutrient inadequacy in infant diets, according to IC consumption, in 3 countries.

	Brazil (n = 108)			US (n = 911)			UAE (n = 72)		
Nutrients	Non-IC Consumers (n = 75)	IC Consumers (n = 33)	*p*-Value ^2,3^	Non-IC Consumers (n = 463)	IC Consumers (n = 448)	*p*-Value	Non-IC Consumers (n = 43)	IC Consumers (n = 29)	*p*-Value
Calcium	19 ^1^	0	**0.005**	17	4	**<0.001**	14	0	0.133
Choline	57	42	0.209	52	47	0.098	77	93	0.618
Folate	28	0	**<0.001**	30	11	**<0.001**	23	24	0.678
Iron	77	6	**<0.001**	50	14	**<0.001**	60	52	0.354
Magnesium	43	6	**<0.001**	41	24	**<0.001**	53	62	0.618
Phosphorus	43	3	**<0.001**	35	23	**<0.001**	28	21	0.447
Potassium	35	6	**0.002**	39	26	**<0.001**	40	34	0.447
Sodium	37	3	**<0.001**	53	67	**<0.001**	51	66	0.750
Zinc	41	3	**<0.001**	25	8	**<0.001**	58	17	**0.003**
Thiamin	31	0	**<0.001**	27	9	**<0.001**	26	3	**0.039**
Niacin	49	6	**<0.001**	29	11	**<0.001**	26	24	0.618
Vitamin A	37	0	**<0.001**	30	20	**0.001**	49	55	0.618
Vitamin D	93	39	**<0.001**	79	71	**0.010**	86	90	0.447

^1^ Numbers expressed as percentage of the population below the recommended intake [43,44,45,46].; ^2^ *p*-values for Fisher’s exact test, adjusted for first discovery rate (FDR).; ^3^ *p*-values < 0.05 are considered significant and are highlighted in bold.

**Table 4:** Nutrient density of observed IC consumer diets versus modeled diets with WGIC and added choline in 3 countries.

	Brazil (n = 33)			US (n = 448)			UAE (n = 29)		
Nutrients ^1^	Current Intakes	Modeled Intakes	*p*-Value ^2,3^	Current Intakes	Modeled Intakes	*p*-Value	Current Intakes	Modeled Intakes	*p*-Value
Protein (g)	3.3	3.5	**<0.001**	2.4	2.5	**<0.001**	2.6	2.7	**<0.001**
Total fat (g)	3.4	3.5	**<0.001**	4.3	4.2	**<0.001**	4.0	4.0	**<0.001**
Carbohydrates (g)	14.2	14.8	**<0.001**	13.4	13.3	**<0.001**	13.2	12.8	**<0.001**
Dietary fiber (g)	0.8	0.9	**<0.001**	0.8	0.9	**<0.001**	0.5	0.7	**<0.001**
Calcium (mg)	120.4	122.5	**<0.001**	78.7	82.9	0.100	79.6	81.9	**<0.001**
Choline (mg)	16.5	28.1	**<0.001**	19.2	21.0	**<0.001**	12.7	16.9	**<0.001**
Folate (μg DFE)	22.5	25.4	**<0.001**	18.1	15.2	**<0.001**	21.2	19.8	**<0.001**
Iron (mg)	2.5	2.9	**<0.001**	2.0	2.6	**<0.001**	1.1	1.3	**<0.001**
Magnesium (mg)	14.3	15.2	**<0.001**	12.5	14.7	**<0.001**	9.1	13.9	**<0.001**
Phosphorus (mg)	73.2	144.9	**<0.001**	48.9	45.6	**<0.001**	53.7	61.5	**<0.001**
Potassium (mg)	161.9	163.4	**0.002**	136.0	130.6	**<0.001**	127.7	115.1	**<0.001**
Sodium (mg)	78.6	80.6	**<0.001**	44.2	41.7	0.310	45.3	48.1	**<0.001**
Zinc (mg)	0.7	1.2	**<0.001**	0.7	1.0	**<0.001**	0.5	0.6	**<0.001**
Thiamin (mg)	0.12	0.1	**<0.001**	0.09	0.08	**<0.001**	0.09	0.09	**<0.001**
Niacin (mg)	1.2	1.0	**<0.001**	1.1	1.0	**<0.001**	0.9	0.9	**<0.001**
Vitamin A (μg RAE)	149.9	144.9	**0.01**	100.1	93.7	**<0.001**	64.2	85.7	**<0.001**
Vitamin D (μg)	1.4	1.6	**<0.001**	0.8	1.1	**<0.001**	0.8	0.9	**<0.001**
**MAR**	0.977	0.980	**<0.001**	0.915	0.931	**<0.001**	0.846	0.894	**<0.001**

^1^ Nutrients were expressed per 100 kcal of intake per day; ^2^ *p*-values for Wilcoxon rank-sum test, adjusted for first discovery rate (FDR); ^3^ *p*-values < 0.05 are considered significant and are highlighted in bold.

**Table 5:** Percentage of infants at risk of micronutrient inadequacy in observed IC consumer diets versus modeled diets with WGIC and added choline in 3 countries.

	Brazil (n = 33)			US (n = 448)			UAE (n = 29)		
Nutrients ^1^	Current Levels	Modeled Levels	*p*-Value ^2,3^	Current Levels	Modeled Levels	*p*-Value	Current Levels	Modeled Levels	*p*-Value
Calcium	0	0	1.000	4	4	1.000	0	0	1.000
Choline	42	6	**0.014**	47	33	**<0.001**	93	76	0.375
Folate	0	0	1.000	11	16	0.175	24	28	1.000
Iron	6	3	1.000	14	11	0.874	52	28	0.346
Magnesium	6	3	1.000	24	17	0.067	62	21	**0.039**
Phosphorus	3	0	1.000	23	32	1.000	21	17	1.000
Potassium	6	6	1.000	26	24	0.874	34	66	0.151
Sodium	3	3	1.000	67	67	1.000	66	62	1.000
Zinc	3	0	1.000	8	5	0.265	17	7	0.873
Thiamin	0	0	1.000	9	9	1.000	3	3	1.000
Niacin	6	6	1.000	11	13	0.874	24	24	1.000
Vitamin A	0	0	1.000	20	22	0.874	55	24	0.151
Vitamin D	39	39	1.000	71	71	1.000	90	79	0.873

^1^ Numbers expressed as percentage of the population below the recommended intake [42,48,50]; ^2^ *p*-values for Fisher’s exact test, adjusted for first discovery rate (FDR); ^3^ *p*-values < 0.05 are considered significant and are highlighted in bold.

## Data Availability

The data presented in this study are available on request from the corresponding author due to privacy reasons.

## Supplementary Materials

The following supporting information can be downloaded at: https://www.mdpi.com/article/10.3390/children12030384/s1, Table S1: Nutritional composition of the recipes used for the diet modelling.
