# Supplementary material for: Impact of Fortified Whole Grain Infant Cereal on the Nutrient Density of the Diet in Brazil, the UAE, and the USA: A Dietary Modeling Study
Source: Children (Basel). 2025 Mar 19;12(3):384. doi: 10.3390/children12030384 (PMC11941295; doi:10.3390/children12030384)
Supplement: Supplementary file 1 [file children-12-00384-s001.zip › children-3496151-supplementary.pdf]

Table S1. Nutritional composition of the recipes used for the diet modelling

Nutritional composition /100g of product as sold	Brazil	US	UAE
Energy (kcal)	340.9	400.0	409
Fat (g)	2.6	8.1	9.7
Protein (g)	8.6	13.3	17.0
Carbohydrates (g)	85.7	64.1	60.4
Fibers (g)	3.4	6.6	5.6
Calcium (mg)	346.7	660.0	551.6
Niacin (mg)	7.2	6.7	6.2
Folic acid (μg)	185.7	46.7	72.6
Potassium (mg)	116.1	347.3	26.1
Phosphorus (mg)	301.8	281.6	514.6
Iron (mg)	33.3	45.0	15.1
Magnesium (mg)	51.0	139.0	107.7
Sodium (mg)	254.8	17.5	235.3
Zinc (mg)	9.5	11.0	6.1
Vitamin A (μg RAE)	1047.0	-	431.3
Vitamin D (μg)	18.0	10.0	8.3
Thiamin (mg)	1.05	0.5	0.73
Choline (mg)	211.9	100.0	100.0
